# Structural and Vibrational Properties of Carboxylates Intercalated into Layered Double Hydroxides: A Joint Computational and Experimental Study

**DOI:** 10.3390/molecules29081853

**Published:** 2024-04-18

**Authors:** Vishal K. Porwal, Erwan André, Antoine Carof, Adolfo Bastida Pascual, Cédric Carteret, Francesca Ingrosso

**Affiliations:** 1 Laboratoire de Physique et Chimie Théoriques UMR 7019, Université de Lorraine and CNRS, F-54000 Nancy, France; 2 Laboratoire de Chimie Physique et Microbiologie pour les Matériaux et l’Environnement UMR 7564, Université de Lorraine and CNRS, F-54000 Nancy, France; 3 Departamento de Química Física, Universidad de Murcia, 30100 Murcia, Spain

**Keywords:** nanoconfinement, layered double hydroxides, hydration, vibrational properties, molecular dynamics simulations

## Abstract

Layered double hydroxides (LDHs) are fascinating clay-like materials that display versatile properties, making them an extremely fertile playground for diverse applications, ranging from bio-compatible materials to the pharmaceutical industry to catalysis and photocatalysis. When intercalating organic and bio-organic species between the inorganic layers, such materials are named hybrid LDHs. The structure–property relation in these systems is particularly relevant, since most of the properties of the materials may be fine-tuned if a comprehensive understanding of the microscopic structure in the interlamellar space is achieved, especially with respect to the reorganization under water uptake (swelling). In this work, we combined experiments and simulations to rationalize the behavior of LDHs intercalating three carboxylates, the general structure of which can be given as $[{\mathrm{Mg}}_{4}{\mathrm{Al}}_{2}{\left(\mathrm{OH}\right)}_{12}]A^{2-}$·${\mathrm{XH}}_{2}$O (with $A^{2-}$ = succinate, aspartate, or glutamate and X representing increasing water content). Following this strategy, we were able to provide an interpretation of the different shapes observed for the experimental water adsorption isotherms and for the evolution of the infrared carboxylate band of the anions. Apart from small differences, due to the different reorganization of the conformational space under confinement, the behavior of the two amino acids is very similar. However, such behavior is quite different in the case of succinate. We were able to describe the different response of the anions, which has a significant impact on the isotherm and on the size of the interlamellar region, in terms of a different interaction mechanism with the inorganic layer.

## 1. Introduction

Minerals, in particular natural clays, have been used as anti-inflammatories, antiseptics, cicatrizers, and cosmetics since prehistory [1]. Their use as active principles (for instance, as gastrointestinal protectors) and in therapeutic activity and aesthetic medicine is widely known. The scientific research in this field has continuously evolved, and the most recent advances have led to the development of new bio-compatible materials inspired by nature and based on synthetic approaches. The knowledge of the molecular structure and of the mechanisms related to the action of clays has pushed even further their potential use in the pharmaceutical industry, in particular for the delivery of drugs and bio-active molecules [2]. Among these materials, layered double hydroxides (LDHs) possess low or null toxicity, good bio-compatibility, and the possibility of leading to controlled release. LDHs comprise inorganic layers having a positively charged surface and packing together, including water and negative ions in the interlamellar region to allow for a neutral environment. Anions of very different nature can be intercalated to obtain hybrid systems, i.e., inorganic, organic, and biological molecules and macromolecules (proteins and DNA) [3,4,5], leading to bio-inorganic hybrid materials [6,7,8,9,10,11,12]. LDHs intercalating molecules with a negative charge born by carboxylate groups, such as deprotonated carboxylic acids, have been systematically synthesized since the 1970s [13], and it has been suggested that they can provide stabilization, transport, and release of drugs, such as ibuprofen, containing such groups [14].

The link between the adsorption of an intercalating anion and the expansion of the interlayer distance depending on the hydration conditions has been extensively documented in the literature, based on X-ray diffraction measurements, for small inorganic anions [15], depollutants [16], and organic dyes [17]. In such systems, the adsorption process and its kinetics are key to optimizing the use of LDHs for water remediation.

**Figure 1:**
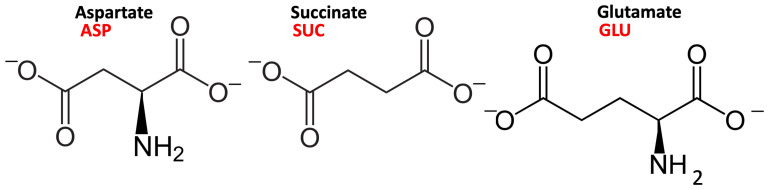
The structure and protonation state of the L-α-aspartate, succinate, and L-α-glutamate anions, with the definition of the abbreviations used in the text.

Among the possible intercalates containing carboxylate groups, amino acids have attracted increasing attention for the production of hybrid materials [18,19,20]. Natural anionic clays might have played a role in concentrating amino acid units, providing a propitious environment for the polymerization leading to polypeptides, the first step towards the origin of life under abiotic conditions [21]. Aspartate, the deprotonated form of aspartic acid, has a deprotonated side chain at neutral and basic pH, and it was present in the first naturally synthesized proteins [22]. It has been suggested that in the mechanism of the anion–surface interaction, mediated by intercalated water molecules, the amino group in amino acids may play a role in anchoring the surface [23]. If we compare the structure of succinate and aspartate, the only structural difference is the absence/presence of an amino group (see Figure 1). Studying the differences in the binding modes of these two molecules containing carboxylate units would, therefore, provide some information for understanding such a mechanism.

It has been shown that the conformations adopted by intercalated biomolecules in solution can either be retained or modified, inducing the unfolding of the secondary, tertiary, or quaternary structure [4], and that intercalated DNA is stabilized compared with bulk water [24]. Given the importance of the structure of biomolecules to deliver their biological function, understanding the way in which nanoconfinement within LDHs affects the configuration, the dynamics, and the reactivity of intercalated species is key to the rational design of hybrid materials.

Experimental work based on X-ray diffraction techniques has provided information on the structural parameters of the inorganic host (distance between the cations within two layers, or interlayer spacing), and vibrational spectroscopy has provided information about the evolution of the vibrational spectrum of the intercalated guests upon hydration [25,26]. However, it is rather complex to achieve a molecular description of the system, especially with respect to the disordered interlamellar space (water structure, anion hydration, etc.). The structure of LDHs intercalating some carboxylic acids [27], amino acids [28], and DNA [24] has been studied by means of molecular dynamics (MD) simulations. Indeed, significant differences have been observed in the structure of the interlamellar region based on the species considered, which motivates us to push further the analysis to rationalize the effect of the anion charge and structure on the local organization. For instance, different amino acids can lead to different orientations with respect to the inorganic layer, and the water structure is strongly dependent on the balance between the interactions with the hydrophilic and hydrophobic groups.

Thanks to the availability of reliable and transferable force fields, such as the widely used Clay force field [29], Monte Carlo and molecular dynamics methods have been successfully applied to analyze the interlayer structure and dynamics [30]. Although remarkable insights were provided by applying these methods to LDHs intercalating carboxylates and amino acids [23,26,27,31,32], we are not aware of previous theoretical work explicitly examining how their different molecular structures can induce differences in the adsorption mechanism inside the material and for different hydration states. As it was already mentioned, from the experimental point of view, the focus so far has been on the synthesis of the material, on proving that the anions are actually intercalated, and on the adsorption kinetics.

The results in the literature strongly hint at the complexity of the local environment in LDHs. In this work, we propose to tackle this challenging problem by means of the interplay between theory and experiments. We propose an approach based on molecular dynamics simulations and on the synthesis and characterization of LDHs intercalating three different anions, choosing different hydration states along the experimental adsorption isotherms and providing an atomistic interpretation of the evolution of the structure–property relationship. In addition to the aforementioned succinate and aspartate, we included a third intercalate, namely, glutamate, which is in all similar to aspartate but displays a longer side chain (Figure 1).

The LDH inorganic precursor derives from the hydrotalcite mineral [33,34,35,36], and its chemical formula can be given as [Mg_4_Al_2_(OH)_12_]CO_3_·4H_2_O. In this compound, the cations are bridged by ${\mathrm{OH}}^{-}$ units coordinated at the octahedral positions, forming sheets packing into a layered structure similar to that of the mineral brucite [37]. The sheets carry net positive charges that must be neutralized by intercalated anions (the carbonate anions in hydrotalcite), and such anions coexist with structural water in the interlayer region. While the hydrotalcite inorganic precursor can be synthesized by using a coprecipitation method, the hybrid LDHs are obtained by anionic exchange from this material [38,39]. According to this procedure, the hydrotalcite precursor undergoes a first anionic exchange with perchlorate anions in ethanaloic medium in order to increase the interlayer spacing [15], as well as to replace the carbonate anion with an easily exchangeable anion. A second anionic exchange is then carried out in water, under nitrogen flow to prevent ${\mathrm{CO}}_{3}^{2-}$ contamination, where the perchlorate is finally replaced by the organic anion of interest. The advantages of this approach are that (i) the first step does not require any precaution regarding carbonate pollution coming from atmospheric CO_2_ and (ii) it provides the same inorganic host (same layer composition and same sheet dimensions) for all hybrid LDHs. Following this synthetic step, in this work, experiments under controlled humidity provided information on the adsorption isotherm and the evolution of the interlamellar space upon hydration, as well as some specific changes in the infrared spectra of the three intercalates. The analysis of our MD simulations delivered a molecular picture unravelling the observed experimental trends.

This paper is organized as follows: In Section 2, we describe the experiments carried out to synthesize and characterize the hybrid systems, as well as the computational setup. The results are discussed in Section 3. Finally, in Section 4, we summarize the major findings of the present work.

## 2. Materials and Methods

### 2.1. Experimental Procedure

#### 2.1.1. Chemicals

MilliQ water purged with nitrogen to remove carbon dioxide and L-α-amino acids were used throughout this work. Di-sodium succinate, sodium aspartate monohydrate, sodium glutamate monohydrate, aluminum chloride hexahydrate (AlCl_3_ · 6H_2_O; ≥99.0%), magnesium chloride (MgCl_2_ · 6 H_2_O; ≥99.0%), and sodium carbonate (Na_2_CO_3_; ≥99.5%) were purchased from Sigma-Aldrich. Perchloric acid (HClO_4_; 60%) was purchased from Riedel-de haën. Sodium hydroxide (NaOH; 1M) was purchased from Corto Erba reagents groups. All products were used as received without any further treatment.

#### 2.1.2. Synthesis of LDHs

Hybrid LDHs were obtained via a double anionic exchange procedure. The starting material was a carbonated Mg_2_Al LDH, synthesized according to the “coprecipitation at constant pH” method previously described [38,39]. In a first exchange, the carbonate anion was replaced with perchlorate (which has a low affinity to the interlayer space) in an ethanol–acid mixture [15]; in brief, 6 mmol of LDH was dispersed in 25 mL of ethanol at 50 °C under nitrogen flow, and a mixture of perchlorate acid (986 μL; 60%) and ethanol (25 mL; 99%) was slowly added to the initial mixture ensuring an excess of perchlorate (the ratio $f=[{\mathrm{ClO}}_{4}^{-}]/2[{\mathrm{CO}}_{3}^{2-}]$ was equal to 1.5). Finally, the mixture was stirred for 1 h under nitrogen flow; then, the slurry was centrifuged, washed with ethanol three times, and dried in air for one night. The anion of interest (succinate, aspartate, or glutamate) was intercalated by a second ion exchange, realized this time under nitrogen flow, in aqueous solution at pH = 10 (to ensure amino acids were present in anionic form). Then, 1 mmol of perchlorated LDH was added to 100 mL of an aqueous solution containing a small excess of the desired anion. The mixture was stirred at 60 °C for 30 min. Afterwards, the mixture was centrifuged, and the solid was dried in nitrogen atmosphere for one night.

#### 2.1.3. Characterization of LDHs

The synthesis of the carbonated LDHs and the successive anion exchanges were monitored by powder X-ray diffraction (PXRD) to follow the evolution of the interlayer spacing and by infrared spectroscopy to ensure the total replacement of the spectral signature of the exchanged anions. Diffraction patterns were recorded with a Panalytical X’Pert Pro MPD diffractometer in reflection geometry by using a tube with Cu radiation (Kα1 = 1.5406 Å), a Ge(111) incident-beam monochromator, 0.02 rad Soller slits, programmable divergence and antiscatter slits (the irradiated area was fixed to 10 × 10 mm), and an X’Celerator detector. Data were collected from finely ground samples with a sample holder spinner and continuous rotation of sample to improve statistical representation of the sample. Infrared spectra were recorded in the 400–4000 ${\mathrm{cm}}^{-1}$ spectral region with a Nicolet 8700 spectrometer equipped with an ATR accessory (GladiATR with diamond crystal; Pike Technologies, Fitchburg, WI, USA) and a DTGS detector. The spectral resolution was set to 4 ${\mathrm{cm}}^{-1}$, and 100 scans were averaged to increase the signal-to-noise ratio. Diffraction patterns for the different LDHs are provided as Supplementary Materials (Figure S1).

#### 2.1.4. Hydration of LDHs

The LDHs’ hydration profiles were studied thanks to water vapor adsorption–desorption isotherms. The experiments were conducted at 298 K by using a MicrotracBEL Belsorp-Max volumetric adsorption analyzer equipped with three pressure sensors (1.33 bar, 13.3 mbar, and 0.133 mbar). Long acquisition times (3 days per isotherm) were required because of the slow equilibrium kinetics. The investigated sample was outgassed under vacuum (residual pressure of $3\times 10^{-6}$ Pa). Volumetric measurements were normalized by considering the molar mass of water-free LDHs (M = 277.1, 235.6, 243.2, and 250.2 g·${\mathrm{mol}}^{-1}$ for ${\mathrm{ClO}}_{4}^{-}$, succinate, aspartate, and glutamate, respectively) in order to derive the number of adsorbed water compound per chloride ion for increasing relative humidity (RH = P/$P_{sat}$ (298 K), with P the water pressure and $P_{sat}$ = 31 mbar the saturation water pressure at 298 K). Mid-infrared diffuse reflectance spectra under controlled water pressure were recorded with a Nicolet 8700 spectrometer, equipped with an MCT detector. The spectra in diffuse reflectance mode were collected by using an environmental cell with a Harrick Praying Mantis. All powder samples were finely ground and dispersed in KBr (2.5 wt%). The relative humidity was controlled by using an in-house dynamic system mixing a dry and a wet $N_{2}$ flow controlled by using a mass flow meter. With such a setup, an RH between 0 and 100% can be generated with an uncertainty of 0.3–0.5%, but the RH range is limited to 0–80 % due to the hygroscopic nature of the KBr matrix.

### 2.2. Simulations

Molecular dynamics simulations were run for LDH materials having three different intercalated anions (L-α-aspartate (ASP), succinate (SUC), and L-α-glutamate (GLU)), under different humidity conditions. The experiments carried out under controlled humidity gave access to the number of water molecules per anion, as well as to the interlamellar space to be used to build the starting configurations corresponding to the experimental conditions. In the case of aspartate and glutamate, experiments, and in particular nuclear magnetic resonance and elemental analysis [40], allowed us to define the protonation state of the amino acid molecules, in agreement with other work carried out under similar pH conditions [26,41,42,43]. The LDH material was described by using the Clay force field (ClayFF) [29], which is based on an ionic (non-bonded) description of the metal–hydroxyl interactions and can be combined with other model potentials. The SPC/E force field was used to simulate water [44], and the Amber force field [45,46] was employed to simulate the intercalated organic anions.

All MD simulations were conducted by using Amber16 [47] and visualized with VMD [48]. The initial cell was chosen to have three layers, to provide three layers and three interlamellar regions in the simulation box, thus improving statistics. After adjusting the interlamellar space to the experimental value for each set of conditions (anion + increasing amounts of water), we replicated the initial cell in the x- and y-directions (with z being the axis orthogonal to the layers). We collect in Table 1 the abbreviations used for the studied systems, the number of molecules per anion (corresponding to the hydration content), and the interlamellar spacing.

The minimization and equilibration protocol and the details about the simulation boxes are fully described in the Supplementary Materials section. In the production run, for each system, MD simulations in parallelepipedal boxes with periodic boundary conditions were run in the NPT ensemble at a temperature of 300 K and a pressure of 1 bar, using Berendsen’s barostat [49], for 10 ns. All quantities measured along the production runs were averaged over the three interlamellar regions of the simulation box.

Infrared spectra were computed by averaging the spectra obtained for each anion by using a 1 ns trajectory. The absorption spectrum of a molecule can be evaluated from MD trajectories by using a formulation stemming from Kubo’s relationship [50]. According to this development, the infrared intensity can be expressed in terms of the Fourier transform of the molecular dipole μ time correlation function as

(1) $$I\left(\omega \right)=\frac{1}{2\pi }Q\left(\omega \right)\int_{-\infty }^{+\infty }dt\;exp\left(-i\omega t\right)<\mu \left(0\right)\cdot \mu \left(t\right)>$$

where Q(ω) is a pre-factor taking into account the quantum nature of the time correlation function [51,52]. An explicit treatment of this factor was not included in our calculation, since this goes beyond the purposes of our analysis. The maximum entropy method was used to compute the Fourier transform [53].

## 3. Results and Discussion

**Figure 2:**
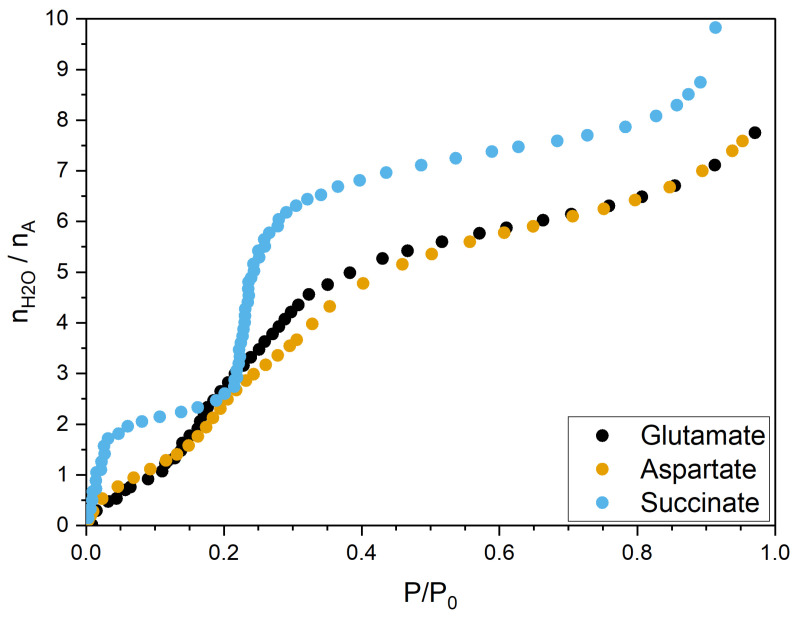
Water adsorption isotherms for LDHs intercalating aspartate, glutamate, and succinate. Relative humidity is given as $P/P_{0}$, with $P_{0}$ corresponding to RH = 100%. Water adsorbed is given as number of water molecules per intercalated anion.

The experiments conducted under controlled humidity provided the adsorption isotherms of the three LDHs intercalating aspartate, glutamate, and succinate, which are displayed in Figure 2. Interestingly, whereas the two curves measured when ASP and GLU are intercalated present an extremely similar shape, when SUC is intercalated, the curve presents one plateau at low humidity and then a very abrupt increase (roughly between two and six water molecules per anion) followed by a smoother increase. The results for ASP are qualitatively consistent with those in Ref. [26].

Following the behavior of the adsorption isotherm for the material intercalating succinate, the interlamellar space changes dramatically between the anhydrous system and the first hydration state considered (see the last column in Table 1), while the LDHs intercalating ASP and GLU undergo a slower transition towards a wider spacing.

**Figure 3:**
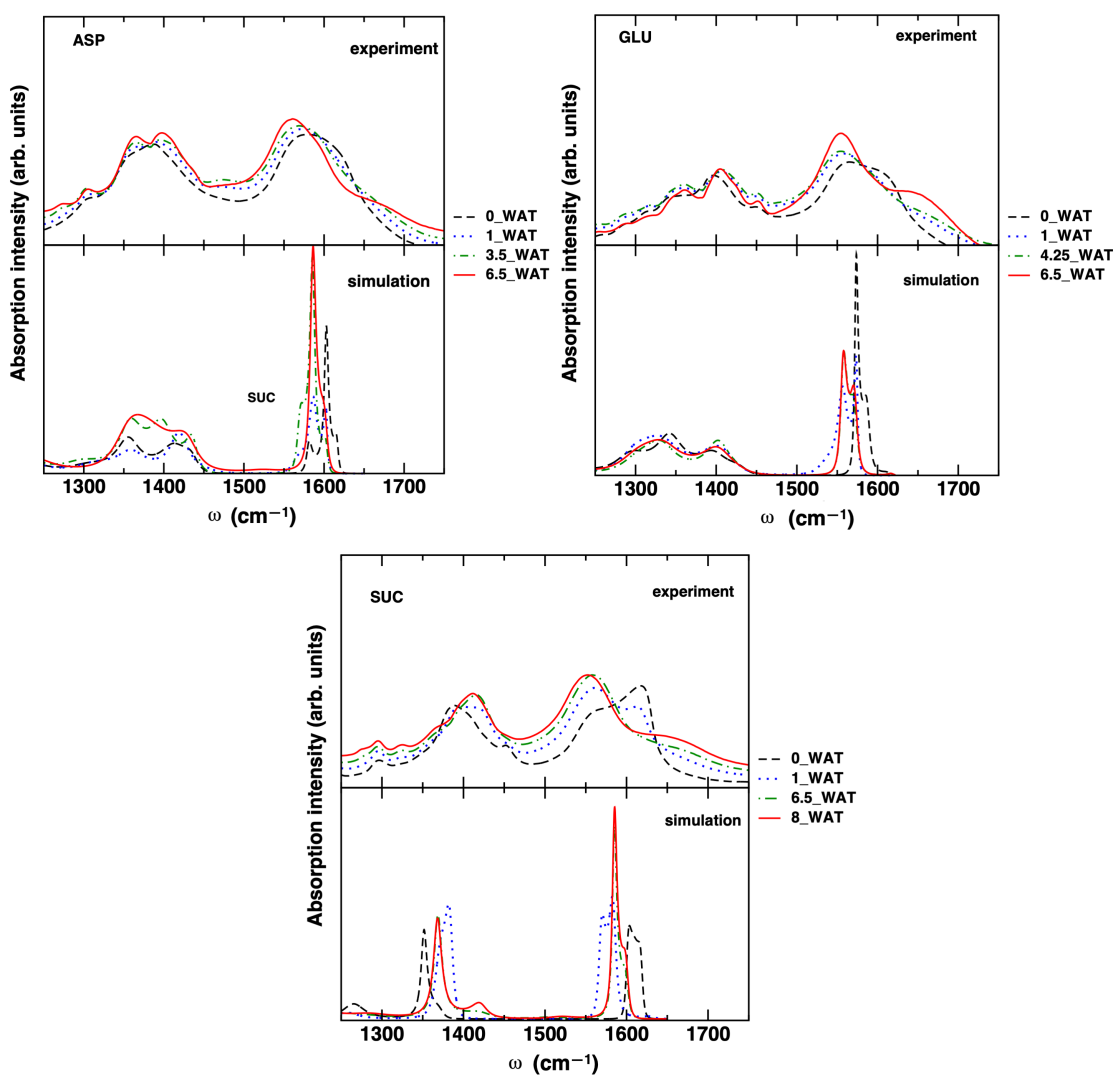
Experimental and simulated carboxylate stretch band of the three intercalated anions for the different hydration states considered for LDHs. The computed bands were shifted (see text).

To unveil the effect of increasing hydration on the molecular properties of the intercalated anions, we monitored the evolution of the carboxylate stretch band in the infrared spectrum. This band, comprising two sub-bands (symmetric (S) and antisymmetric (AS) stretches, at about 1400 and 1600 ${\mathrm{cm}}^{-1}$, respectively) is sensitive to the environment (e.g., hydrogen bonds with water, with the hydroxyl groups on the surface, and among anions) and to coupling with other modes, as the N-H bend for the amino acid systems. It also has the advantage of not overlapping with IR bands of other portions of the system (water and OH groups). Our experimental and computational results are reported in Figure 3.

For the computed spectra, the assignment of the bands was confirmed by analyzing the Fourier transform of the atomic velocities of the C and O atoms of the carboxyl groups and of the N atoms (not reported for the sake of simplicity; see Ref. [54] for further details). The computed bands were shifted by −200 ${\mathrm{cm}}^{-1}$ in all systems to match the experimental result. Such a shift is not surprising, since we neglected the pre-factor in Equation (1) and we used a standard force field without specific reparametrization of the intramolecular potential. The value of the shift is consistent with what some of us found for the C=O stretch of similar molecules in water solution [54,55] by using different simulation methods (molecular mechanics-based MD, Born–Oppenheimer MD, and hybrid quantum mechanics–molecular mechanics dynamics) and Equation (1). The poor description of the band shape for the AS stretch band is also related to some limitations of the computational approach. This particular aspect would require significant efforts [56] that would be beyond the scope of the present work. On the other hand, the value of the splitting between the S and AS (sub-)bands is in fairly good agreement with the experiments, as it can be observed in Table 2, especially for ASP and GLU. In addition, the trend with increasing hydration qualitatively matches the experimental trend, as does the shape of the S stretch band, which is quite wide and most likely results from a superposition of multiple bands for ASP and GLU, whereas it is narrower for SUC. Such band may be affected by coupling with the vibrations of the −${\mathrm{CH}}_{3}$ groups, as some of us showed in Ref. [55] for the aspartate anion in water solution, a coupling which does not occur in the case of succinate.

In the systems intercalating ASP and GLU, we see the AS band progressively red-shifting, while the S band is much less affected. In the case of ASP, the measured bands are in good agreement with the published results in Ref. [26]. It is worth noting that for these two systems, a shoulder appears in the highest hydration state in the higher-frequency region of the experimental data. In past work on the carboxylate bands of ASP in water [55], we found that this shoulder is present when explicit water molecules are included in the solvation shell of the anion, whereas it is not present when a continuum model is considered. We were able to show that the shoulder is due to the coupling of the AS stretch of the carboxylate unit with the bend vibration of water. In the present work, the molecular mechanics force field used is most likely not able to catch this fine feature. We can safely assume that the presence of the shoulder acts as a probe of a more hydrated environment of the C=O oscillator. The band gap for the system intercalating SUC is overestimated compared with experiments but the trend is qualitatively the same. In moving from the anhydrous system to the hydrated systems, the S stretch band is blue-shifted and the AS band red-shifted. From this analysis, we can conclude that our simulations are able to catch the most important features in the spectral evolution of the carboxylate band as the water content increases in the LDH material and that the bands of the system intercalating SUC are more significantly affected than in the cases of ASP- and GLU- intercalated LDHs.

To analyze this behavior, we performed a study of the evolution of the local structure in the galleries upon hydration. For the system intercalating ASP, it has already been discussed in the literature that the anions reorient themselves when the water content increases, starting from a situation in which they lie parallel with respect to the layer in the anhydrous system [26,31]. In studies considering comparable values of the interlamellar spacing in LDHs, a monolayer arrangement of the anions in the galleries has been hypothesized, in agreement with what we observe [43,57,58,59].

In order to follow anion reorientation, similarly to Ref. [26], we monitored the θ angle formed by a vector joining the two carbon atoms of the carboxylate groups with respect to the normal to the surface. To rationalize our results, we present them in terms of populations of orientational states, computed as percentages. We count anions in a parallel orientation when θ is larger than $80.0^{\circ }$ and in an intermediate/perpendicular orientation otherwise. The results are reported in Table 3.

**Figure 4:**
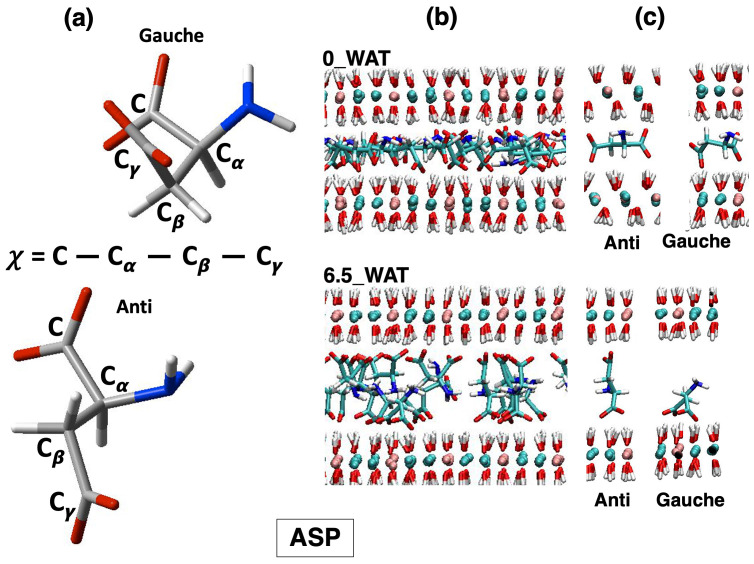
LDH intercalating aspartate anions. (**a**) Structures of the anions in the relevant conformations observed on average along the MD simulations of LDHs and definition of the dihedral angles computed to describe such conformations. (**b**) Snapshots illustrating the organization of the anions in the anhydrous system and in the most hydrated state. (**c**) Most relevant configurations observed for the anions in the interlamellar space and in the corresponding hydration state.

**Figure 5:**
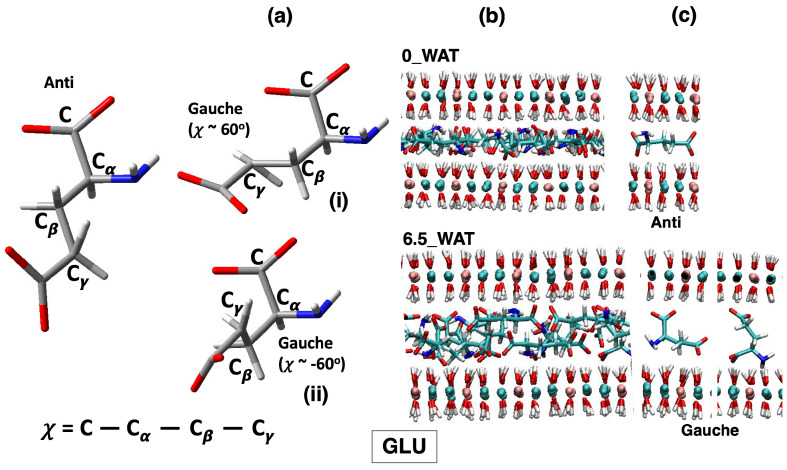
LDH intercalating glutamate anions. (**a**) Structures of the anions in the relevant conformations observed on average along the MD simulations of LDHs and definition of the dihedral angles computed to describe such conformations. (**b**) Snapshots illustrating the organization of the anions in the anhydrous system and in the most hydrated state. (**c**) Most relevant configurations observed for the anions in the interlamellar space and in the corresponding hydration state.

**Figure 6:**
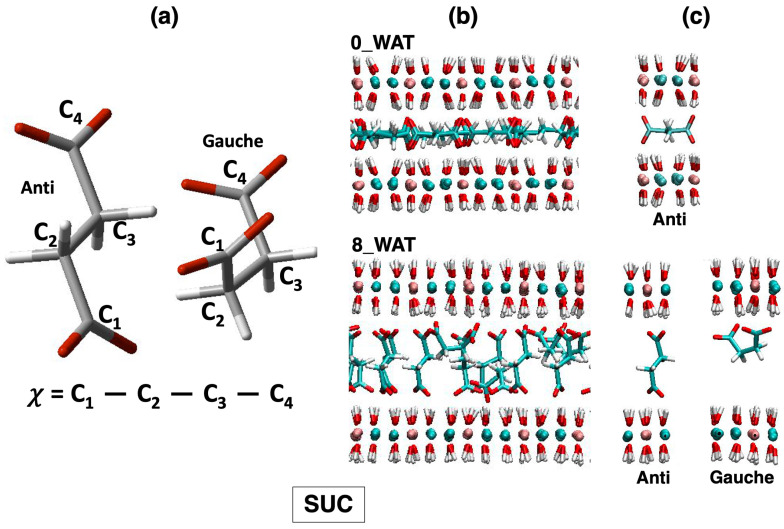
LDH intercalating succinate anions. (**a**) Structures of the anions in the relevant conformations observed on average along the MD simulations of LDHs and definition of the dihedral angles computed to describe such conformations. (**b**) Snapshots illustrating the organization of the anions in the anhydrous system and in the most hydrated state. (**c**) Most relevant configurations observed for the anions in the interlamellar space and in the corresponding hydration state.

All systems undergo a reorientation with the increase in the amount of water in the interlamellar space, and for each system, we observe an intermediate state for which the number of anions lying horizontally decreases drastically, followed by hydration states for which a slight increase in this population is seen. Therefore, this descriptor alone cannot account for the differences observed in the three systems. As a matter of fact, the underlying interpretation is still blurred by the fact that the choice of a global descriptor of reorientation, such as the vector joining the C atoms of the carboxylate groups, may hide some relevant degrees of freedom, particularly those related to the conformational space of the anions. To complete this analysis, we monitored the dihedral distributions (reported as Supplementary Materials, Figures S3–S5) of the C-C-C-C dihedral in SUC and the C-$C_{\alpha }$-$C_{\beta }$-$C_{\gamma }$ dihedral in the amino acids, defined in Figure 4, Figure 5 and Figure 6, panel (a), allowing us to recognize the more stable conformers, anti and gauche [55,60,61].

**Figure 7:**
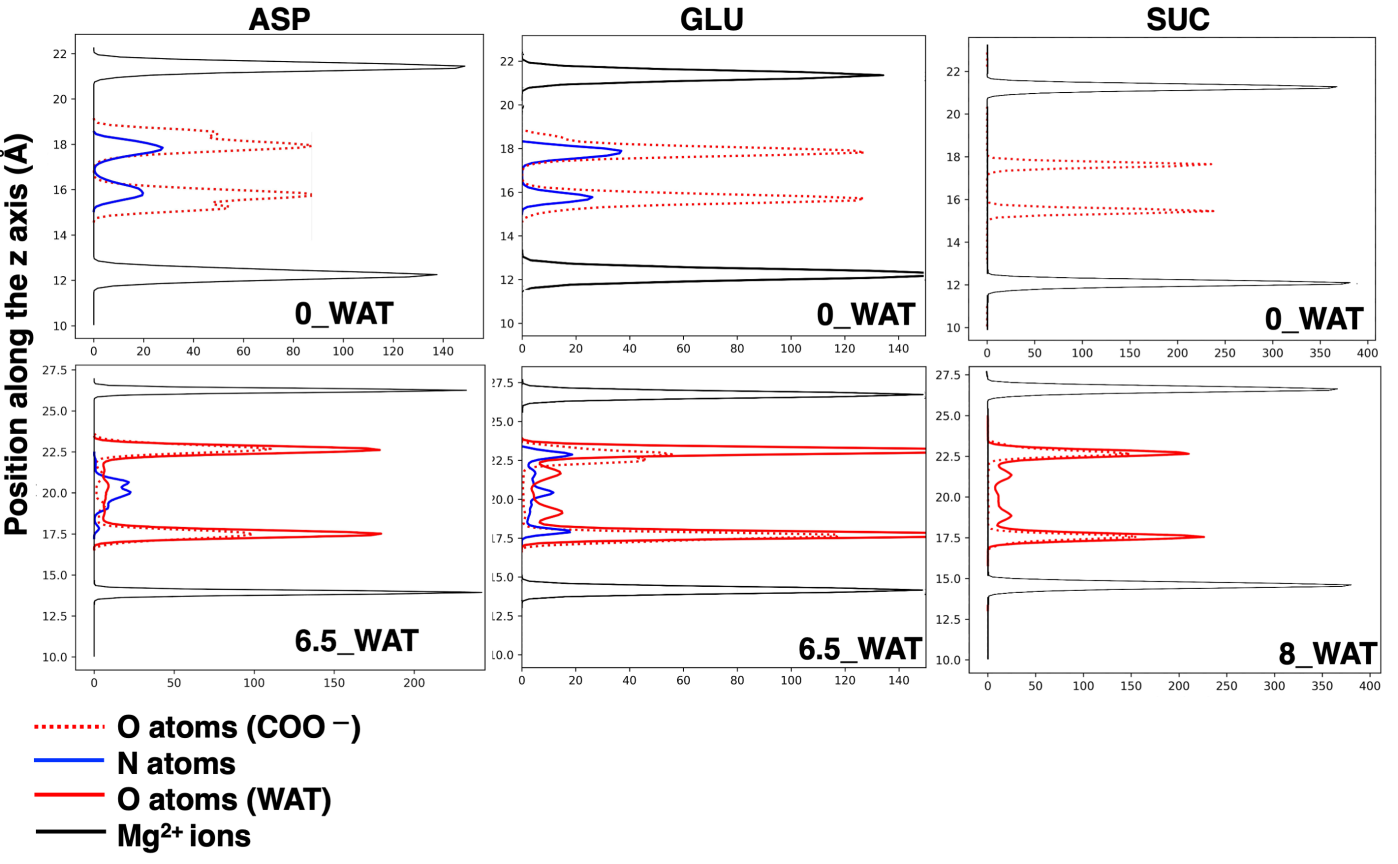
Number densities along the axis perpendicular to the surfaces of relevant atoms in the interlamellar space, shown for the anhydrous systems (**top** plots) and for the higher hydration states (**bottom** plots) for the materials intercalating ASP, GLU, and SUC (from **left** to **right**). The distributions of the z positions of the Mg^2+^ cations are displayed as a reference.

In the case of aspartate, both the anti and gauche conformations are present in all hydration states. An illustration of the change in the global organization of the interlamellar space is depicted in panel (b) of Figure 4, while some structures extracted from those snapshots show ASP in anti and gauche conformations interacting with the surface. These illustrations also show that such interaction is driven both by the carboxylate oxygens and by the N atom, which is an important confirmation of the findings reported in Ref. [23]. To shed more light on this observation, we computed the number density of N atoms along the z-axis, orthogonal to the surface, as shown in Figure 7. In order to facilitate the description, we chose to report in Figure 7 our results for the anhydrous systems as well as for the most hydrated states, and the collection of all our results is provided as Supplementary Materials, Figure S2.

The carboxylate oxygen atoms and the nitrogen atoms from the amino groups sit next to the surface in the anhydrous system and in the lower hydration state. However, our analysis reveals that most of the N atoms are found in the middle of the interlamellar region in the highest hydration state (6.5_WAT), as the snapshot in Figure 4b displays. When ASP is in anti, the two carboxylate groups interact with two different surfaces. Conversely, when it is in gauche (a more compact configuration) they both lie next to the same surface, whereas the −${\mathrm{NH}}_{2}$ group can reorient itself toward the middle of the interface.

As in the case of ASP, for GLU, both the anti and gauche conformations are present in all hydrated systems, but in the anhydrous system, only the anti conformer is present. In addition, in the case of aspartate, the proximity of the amino group to the carboxylate group at the $C_{\beta }$ carbon and the resulting steric hindrance only allow for one gauche orientation (χ∼−$60^{\circ }$). However, the presence of a longer side chain in glutamate allows for two possibilities (χ = −$60^{\circ }$ and χ = $60^{\circ }$), presented as structures (i) and (ii) in Figure 5a and as extractions from MD simulations, interacting with the surface, in Figure 5c. In all cases, the N atoms interact strongly with the surfaces, as can be retrieved by the analysis of the number density along z, in the middle panel of Figure 7. In the highest hydration state, part of the N atoms are also present in the middle of the interlamellar space, but the population interacting with the surface is still present, unlike the LDH intercalating ASP. On the one hand, the anions lying perpendicularly to the surface display N in the center of the interlayer; on the other hand, those in gauche conformations may have it either interacting with the surface or in the middle of the interlayer (see illustration in panel (c) of Figure 5). We can conclude that notwithstanding the similar structure of the two amino acids, the different length of the side chain triggers a different exploration of the conformational space under confinement, as it was observed by using MD to study these anions intercalated in the same material but in a different protonation state [32]. To complete the analysis of the number density (ASP and GLU), we note that the O atoms of the water molecules are found, in all cases, in close proximity to the carboxylate O atoms, a finding that is fully consistent with the reported affinity of water for the carboxylate groups, in LDHs as in water solutions [27,28,55,60]. In the higher hydration state, some water molecules are also present in the middle of the interlayer.

Finally, in the system intercalating SUC, the gauche configuration is not observed in the anhydrous system and in the lowest hydration state, but it gets slightly more populated in the highest hydration states (corresponding to a higher population of anions with parallel orientation; see an illustration in Figure 6c). However, the majority of the anions switch from an anti configuration, with the molecule horizontal to the surface (low hydration and narrower interlayer), to an anti configuration with perpendicular orientation as soon as the uptake of water has allowed for a wider interlamellar spacing. This is the key difference in the response of succinate to hydration, compared with the amino acids, since the latter can adapt to the changes in the confined space with additional degrees of freedom, enhanced by the interaction of the amino group with the surface and with water. As for the amino acids, water molecules sit next to the carboxylates of SUC (right panel of Figure 7), but some of them appear inside the gallery at higher hydration. The general picture that we draw is in agreement with the behavior of SUC in Zn/Al- and Al/Cr-based LDHs, in which a contraction of the interlayer is observed (which is interpreted as a reorientation of the anion) under thermal treatment [57].

The collection of the results that we presented, as well as some observations based on visual inspection of the trajectories, allow us to introduce some comments on the binding modes of the carboxylate moieties, by analogy with their binding modes in the presence of a metal [62,63,64,65]. Surely, such units are key to driving the interaction between molecules containing carboxylate units and the layer, as it has been clearly shown by extracting a one-dimensional electron density map from X-ray diffraction experiments [66]. In our case, the dramatic change in the orientational state of SUC corresponds to a change from bidentate bridging binding (each O of one carboxylate interacts with a different surface) to bidentate chelating (both O interacting with the same surface). When ASP or GLU are intercalated, both types of binding are present under all hydration conditions, and an abrupt change of interaction modes does not take place.

In the 1980s, Deacon and Phillips provided an extensive investigation of the relative position of the anti-symmetric and symmetric stretch bands for complexes based on acetate and trifluoroacetate complexes [62]. The AS-S band gap was correlated with the type of binding; compared with the free anion, a larger band gap band would be observed for monodentate binding, a smaller band gap for bidentate chelating, and a similar band gap for bidentate bridging modes. Those criteria have been extensively used in the literature to assign the type of binding of a wide variety of compounds. However, the authors themselves warned against applying this classification without caution, especially in the case of complex systems and for different experimental conditions. Among more recent studies, we chose to report two that represent the kind of controversy that is still related to the correlation between the band gap and the binding mode. In 2015, Sutton et al. [65] performed a computational study of complexes of acetate and formate with different cations in an aqueous environment. For these carboxylates, the predictions by Deacon and Phillips were fulfilled, but the main correlation was between the band gap and the geometrical parameters of the carboxylate groups. As a matter of fact, the distortion of the C-O bond and of the O-C-O angle upon complexation would explain the evolution of the band gap with the different modes of binding. On the other hand, in 2010, Martinez et al. [64] performed an experimental investigation of the AS-S band gap for manganese complexes of carboxylates having a more complicated structure, and their conclusions do not support the prediction of the evolution of the band gap with binding modes, but they do prove that the experimental conditions are key to correctly assigning the C-O stretch band, since a change in the coordination sphere of the metal can be observed.

Considering that in our case, the interaction takes place with a charged surface (and not with a metal center), that coupling with the motions involving the amino/methyl groups occurs in ASP and GLU, and that a final answer on the correlation between binding modes and the S vs. AS band gap does not clearly emerge from the literature, the results that we obtained hint at a stronger effect on the band gap and shape in the case of SUC. The band gap decreasing with the increase in hydration, for all systems, is both due to the conformational reorganization of the anions and their interactions with water.

**Figure 8:**
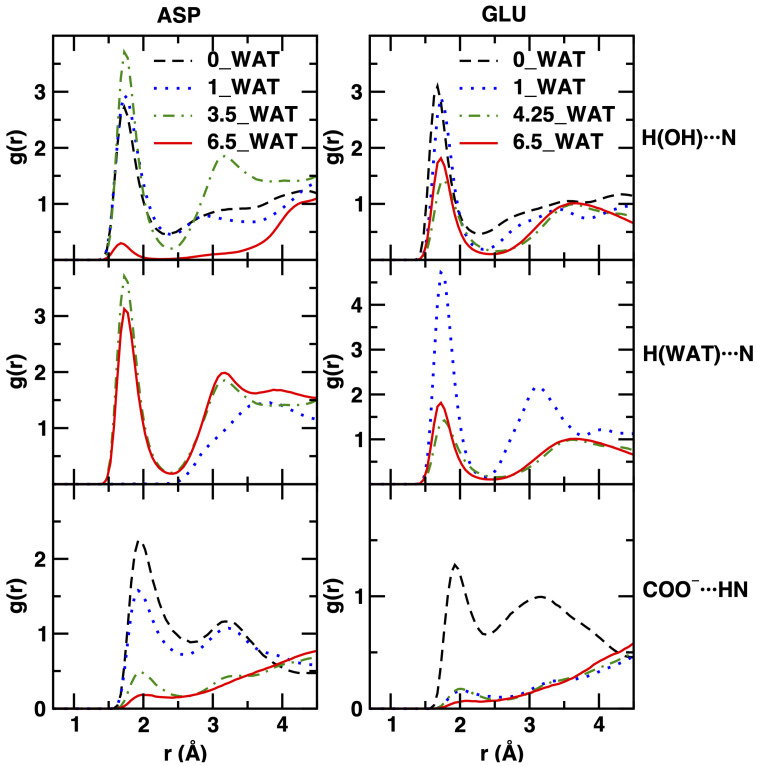
Radial distribution functions for atom–atom interactions, displayed for distances consistent with the size of the interlamellar space. On the left column, we show results for the system intercalating ASP and on the right column for that intercalating GLU. From top to bottom: interactions between the H atom of the hydroxyl groups on the surface with the N atom of the intercalates; interactions of the H atom of water with N; interactions between the oxygen atoms of the carboxylate unit of one intercalate with the H atom at the N site of another one.

To shed more light on the interactions of the anions with the local environment, including water and the surface, we conclude our discussion by presenting the results of the computed radial distribution functions (RDFs) for some relevant atom–atom interactions. For the LDHs intercalating ASP and GLU, we observed interesting changes with the increase in the water content, as displayed in Figure 8.

The interactions between the amino acids and the layers, monitored by using the RDFs of the H atom of the hydroxyl groups of the surfaces and the N atom of the anions, generally decrease when the water content (and thus the interlamellar space size) increases; in agreement with the density increase of N atoms in the center of the gallery, this effect is stronger in the case of the aspartate intercalate. In addition, for this system, the 3.5_WAT state seems to suggest slightly stronger interactions. We recall that in this hydration state, we observed an intermediate behavior with respect to the orientation of the anions. Regarding the interactions with water, we note that they stay more or less the same, with the exception of the 1_WAT system intercalating GLU (as above, this is the case for which an intermediate reorientational behavior was observed).

The most intriguing result is related to the evolution of the anion–anion interactions, through the (weak) hydrogen bond interactions between the charged O atoms of the carboxylate groups and the acidic H atom attached to the N atom of the amino acids. Two very structured peaks are observed here within the distances corresponding to one interlamellar space (up to 4–5 Å). This interaction, well characterized in the anhydrous system and in the 1_WAT systems, gradually fades away with the increase in hydration, as the first peak loses intensity and structuring. Our results are thus consistent with the hypothesis that lower hydration leads to much stronger COO^−^⋯HN interactions, bringing the amino acid units to the proximity that might finally lead to chemical reactivity (i.e., polypeptides formation) in natural systems [23,26,67]. Differences between the behavior of the aspartate and glutamate anions upon the increase in hydration are mostly observed in the shape and in the evolution of the second, much broader peak. This peak disappears at higher hydration conditions in the case of the LDH intercalating aspartate, while it not only stays but becomes more structured in the case of glutamate. However, in both cases, the evolution of the RDF shapes can be interpreted as a sign of weaker proximity along the COO^−^⋯HN direction, which is more sudden in the case of aspartate and more regular in the case of glutamate (especially for the first peak). We recall here that this interpretation needs to be kept at a qualitative level, since we are applying definitions that are based on spherical symmetry (i.e., the RDF) to a system with a different topology.

**Figure 9:**
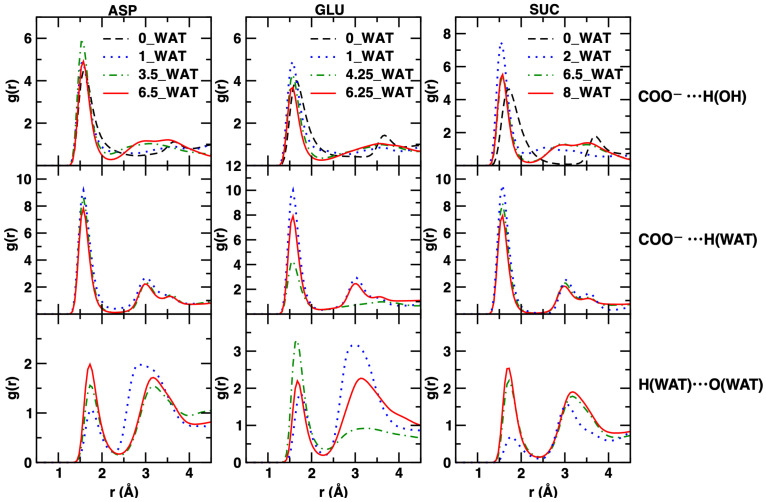
Radial distribution functions for atom–atom interactions, displayed for distances consistent with the size of the interlamellar space. In the left column, we show results for the system intercalating ASP; in the middle column, those for the system intercalating GLU, and in the right column, those for the system intercalating SUC. From top to bottom: interactions between the H atom of the hydroxyl groups on the surface with the O atoms of the carboxyl groups of the intercalates; interactions of the H atom of water with the O atoms of the carboxyl groups of the intercalates; interactions between the H and the O atoms of water molecules.

Our inspection of the interactions of the carboxylates, both with the surface and with the water molecules, and of water-water interactions are reported in Figure 9 for the three anions considered.

Our results converge in pointing to a strong interaction established between the carboxylate groups of all systems and with both the water molecules and the surface. The structure of water around those groups is generally very similar in all cases, even under lower hydration. This can be safely interpreted as a fingerprint of a local hydrogen bond network that is well established in all situations. However, it is interesting to note that in the case of SUC, a drastic change is observed in the first peak of the RDF characterizing the intermolecular H⋯O interactions in water. In the 2_WAT system, such peak is smaller than in the case of the most hydrated states. With the help of our results for the number density along z, we can estimate that the local network of water hydrogen bonds is only recovered when water molecules start populating the middle region of the interlayer (4.25_WAT and 6.5_WAT), while it is weakened if water is only present next to the surface (2_WAT).

## 4. Conclusions

In this work, we presented a joint experimental and molecular dynamics study of the properties of LDH hybrid materials intercalating aspartate, succinate, and glutamate anions. In particular, we modeled the anhydrous systems and three different hydration states corresponding to the experimental conditions under which the measurements were carried out.

We compared the experimental and simulated carboxyl stretch bands, finding that the S and AS band gap and its evolution with increasing hydration are nicely accounted for by our model. A stronger effect is observed in the case of succinate, whereas a decrease in the band gap (but smaller effects on the band shape) are seen for aspartate and glutamate. We were able to relate the strong effect measured for succinate with a different reaction to increasing the water content in the material. SUC reorients itself completely, from lying horizontally with respect to the surfaces to lying perpendicularly, mostly keeping an anti conformation and moving from a bridging bidentate mode to bridging chelating with respect to the surface. The presence of the −${\mathrm{NH}}_{2}$ group, an additional source of interaction with the surface and with water, and the more complex conformational space explored by ASP and GLU, lead to partial reorganization in the interlayer and to the presence of both anti and gauche conformers.

To summarize, we were able to show that relying on a single descriptor (the angle formed by the vector joining the C atoms of the ${\mathrm{COO}}^{-}$ groups and the z-axis) does not provide conclusive answers on the reorganization of the intercalated species, since this is not the only degree of freedom in non-rigid structures such as those of the three anions considered. The picture arising from our investigation is in agreement with the observed shape of the hydration isotherms, which are similar and featureless for ASP and GLU, whereas for SUC, the curve presents a plateau after the first steps of the water uptake and then a very steep rise, followed by a much slower increase. Once the switch in the two populations of succinate is made, the gallery can more easily pack water molecules, since the perpendicular orientation of the anions and their simple molecular structure allow for more room. On the other hand, the presence of an additional source of intermolecular interactions (with the surface, with water, and with the other anions) and the richer configurational space in ASP and GLU lead to a smoother, more regular change when increasing the water content.

The analysis of radial distribution functions, restricted to distances corresponding to the size of the interlamellar space, as well as the number distribution of significant atoms along the z-axis (perpendicular to the surface) provided information on the most important intermolecular interactions that are active in the confined systems, on the position of the polar groups, and on how they evolve with increasing water content. The amino group of the amino acids can act as a hydrogen bond acceptor, from the surface and from water, but under anhydrous conditions, it acts as a hydrogen bond donor to the carboxylate group of a nearby anion. The latter situation becomes increasingly less favorable with increasing hydration, a phenomenon that was already mentioned in the literature and claimed to be a possible path to the synthesis of peptide bonds in abiotic conditions and low water content [23,26].

Our findings show that combining computational and experimental work can significantly increase our knowledge of the microscopic structure of hybrid LDHs, making further steps toward a rational design of their properties. Increasing attention is being paid in the literature to hybrids containing carboxylate anions for applications in the electro- and photo-catalysis domain [68,69,70,71]. The role of these groups may impact the reaction kinetics by acting as a Lewis base [69]. Preliminary, non published results by some of us confirm the role played by the carboxylate groups in modulating the interaction of intercalates with the surface, which can be strongly affected by the electronic state of the molecules, as it was suggested by Xiao et al. [72]. This synergistic approach can lead to a constructive interplay between simulation and experiment, trigger the improvement of the models through fine tuning based on measured properties, and promote novel analytical and synthetic discoveries in the field of hybrid materials.

## Figures and Tables

**Table 1:** Definition of the simulated systems.

Systems	No. of Water Molecules per Anion (Hydration Content)	Interlamellar Spacing (Å)
LDH intercalating ASP		
0_WAT	0 (0%)	9.06
1_WAT	1 (10%)	9.21
3.5_WAT	3.5 (30%)	11.21
6.5_WAT	6.5 (80%)	12.10
LDH intercalating GLU		
0_WAT	0 (0%)	9.08
1_WAT	1 (10%)	11.57
4.25_WAT	4.25 (30%)	12.35
6.5_WAT	6.5 (80%)	12.35
LDH intercalating SUC		
0_WAT	0 (0%)	8.87
2_WAT	2 (10%)	11.38
6.5_WAT	6.5 (30%)	11.98

**Table 2:** Shifts between the asymmetric and the symmetric stretch carboxylate bands: comparison between experiments and simulations.

Systems	Experimental Band Gap (${\mathrm{cm}}^{-1}$)	Computed Band Gap (${\mathrm{cm}}^{-1}$)
LDH intercalating ASP		
0_WAT	196	210
1_WAT	190	209
3.5_WAT	188	197
6.5_WAT	178	197
LDH intercalating GLU		
0_WAT	211	213
1_WAT	198	185
4.25_WAT	198	192
6.5_WAT	175	187
LDH intercalating SUC		
0_WAT	230	257
2_WAT	145	210
6.5_WAT	145	200
8_WAT	145	200

**Table 3:** Orientational states for the anions with increasing hydration.

Systems	Parallel Orientation (%)
ASP	
0_WAT	55
1_WAT	39
3.5_WAT	9
6.5_WAT	32
GLU	
0_WAT	86
1_WAT	4
4.25_WAT	15
6.5_WAT	16
SUC	
0_WAT	100
2_WAT	0
6.5_WAT	15
8_WAT	26

## Data Availability

The diffraction spectra of the materials, further details about the computational procedure, the distribution of the dihedral angles describing the conformations of the anions, and the plots of the number densities computed for all hydration states are provided as Supplementary Materials.

## Supplementary Materials

The following supporting information can be downloaded at: https://www.mdpi.com/article/10.3390/molecules29081853/s1. References [29,44,45,46,47,48,49,73,74,75] are cited in Supplementary Materials.
