# Supplementary material for: Structural and Vibrational Properties of Carboxylates Intercalated into Layered Double Hydroxides: A Joint Computational and Experimental Study
Source: Molecules. 2024 Apr 18;29(8):1853. doi: 10.3390/molecules29081853 (PMC11054486; doi:10.3390/molecules29081853)
Supplement: Supplementary file 1 [file molecules-29-01853-s001.zip › molecules-2964820-supplementary.pdf]

Supporting Information for: Structural and Vibrational Properties of Carboxylates Intercalated into Layered Double Hydroxides: a Joint Computational and Experimental Study

Vishal K. Porwal,[†] Antoine Carof,[†] Erwan André,[*,‡] Adolfo Bastida Pascual,[¶] Cédric Carteret,[‡] and Francesca Ingrosso[*,†]

†*Université de Lorraine and CNRS, Laboratoire de Physique et Chimie Théoriques UMR 7019*

‡*Université de Lorraine and CNRS, Laboratoire de Chimie Physique et Microbiologie pour les Matériaux et l'Environnement UMR 7564*

¶*Departamento de Quìmica Fisica, Universidad de Murcia*

E-mail: erwan.andre@univ-lorraine.fr; francesca.ingrosso@univ-lorraine.fr

The diffraction spectra of the materials, details about the computational procedure, the distributions of the dihedral angles describing the conformations of the anions, the plots of the number densities computed for all hydration states are provided in the following.

Experimental Diffraction Spectra

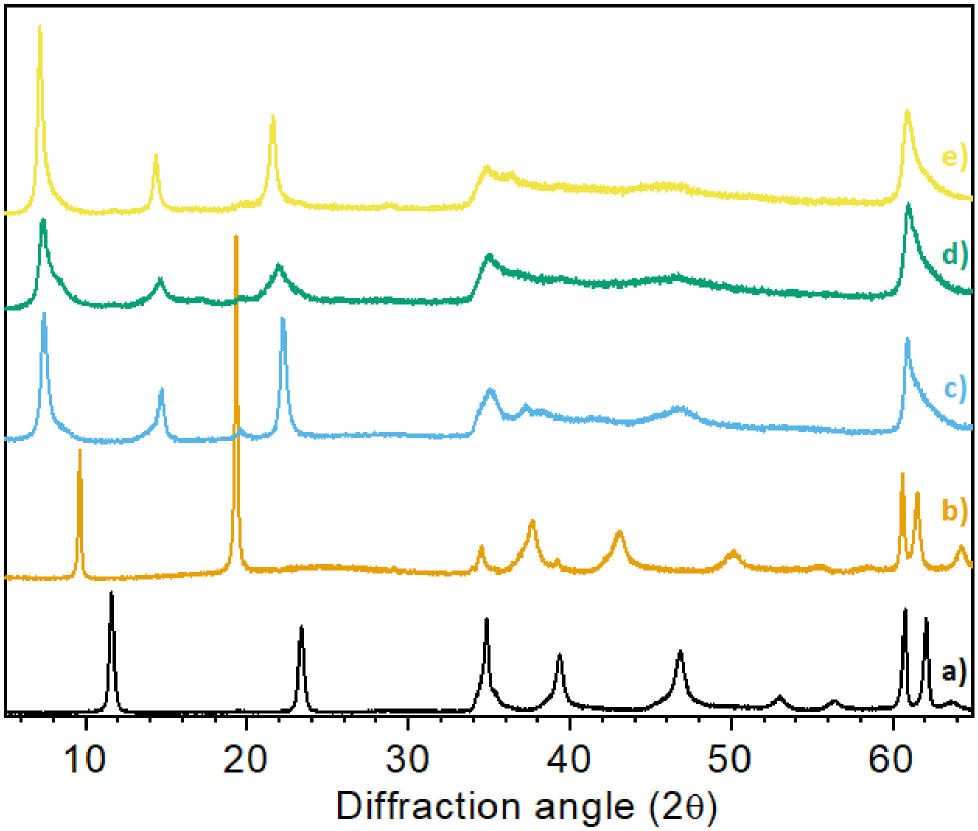

Figure S1: Experimental diffraction patterns of Mg_2Al LDH intercalated with a) CO_3^{2-}, b) ClO_4^-, c) Succinate (SUC), d) Aspartate (ASP), e) Glutamate (GLU).

Computational Details

Molecular dynamics simulations were run for LDH materials having three different intercalated anions (aspartate (ASP), succinate (SUC) and glutamate (GLU)), each for different humidity conditions. Experiments were carried out under controlled humidity, giving access to the experimental number of water molecules per anion as well as to the interlamellar space to be used to build the starting configurations corresponding to the experimental conditions. In the case of aspartate and glutamate, the experimental analysis allowed us to define the protonation state of the amino acid molecules. The LDH material was described using the Clay force field (ClayFF),[1] which is based on an ionic (non-bonded) description of the metal-oxygen interactions and has a flexible force field framework that can be combined with other potentials. The SPC/E potential was used to simulate water[2] and the Amber force field[3,4] was employed to simulate the intercalated organic anions.

All MD simulations were conducted using Amber 16 and Amber Tools[5] and visualized with VMD.[6] The initial cell of Mg/Al 2:1 LDH has a three-layer structure, to provide three layers and three interlamellar regions in the simulation box. After adjusting the interlamellar space to the experimental value for each set of conditions (anion + increasing amounts of water, see Table 1), we replicated the initial cell in the x,y directions (z being the axis orthogonal to the layers). Experiments provided the number of water molecules per anion and the value of the interlamellar space based on their work on the material as well as the protonation state of the amino acids anions.

For each system and each considered hydration state, energy minimization was performed using the steepest descent method and conjugate gradient algorithms.[7] The Al^{3+} and Mg^{2+} cations were restrained with force constant 100 kcal/mol/$Å^2$ and the SHAKE algorithm was also used to keep the distance constant between hydrogen atoms and heavy atoms connected by covalent bonds.[8] A preliminary step for equilibration was achieved by simulated annealing: the system was heated (up to 2500K) and cooled (to 300K) for 5.5 ns by gradually changing the temperature in the NVT ensemble, using Andersen's temperature coupling scheme.[9]

Table S1: Parameters defining the box size and the water content of the systems considered.

**Systems**	**No. of water molecules / anion**	**Interlamellar space (Å)**	**Box size (Å)**		
			$\mathbf{L}_x$	$\mathbf{L}_y$	$\mathbf{L}_z$
**LDH intercalating ASP^{2-}**					
0_WAT	0 (0%)	9.06	31.707	54.918	27.180
1_WAT	1 (10%)	9.21	31.707	54.918	27.630
3.5_WAT	3.5 (30%)	11.21	31.707	54.918	33.630
6.5_WAT	6.5 (80%)	12.10	31.707	54.918	36.300
**LDH intercalating GLU^{2-}**					
0_WAT	0 (0%)	9.08	31.707	54.918	27.240
1_WAT	1 (10%)	11.57	31.707	54.918	34.710
4.25_WAT	4.25 (30%)	12.35	31.707	54.918	37.050
6.5_WAT	6.5 (80%)	12.35	31.707	54.918	37.050
**LDH intercalating SUC^{2-}**					
0_WAT	0 (0%)	8.87	31.707	54.918	26.610
2_WAT	2 (10%)	11.38	31.707	54.918	33.900
6.5_WAT	6.5 (30%)	11.98	31.707	54.918	36.300
8_WAT	8 (80%)	12.06	31.707	54.918	36.300

During this step, the positions of the OH^- groups, Al^{3+} and Mg^{2+} were constrained with a 500 kcal/mol/Å^2 force constant. A 1 ns trajectory was then run in the NPT ensemble at a temperature of 300 K and a pressure of 1 bar, using Berendsen's barostat.[10] Following this final equilibration step, the constraints on the hydroxyl groups were released, and a force constant of 10 kcal/mol/Å^2 was kept for Al^{3+} and Mg^{2+}. Data analysis was performed on a subsequent NPT simulation (10 ns) using the same parameters as those of the previous NPT step.

Local Structure

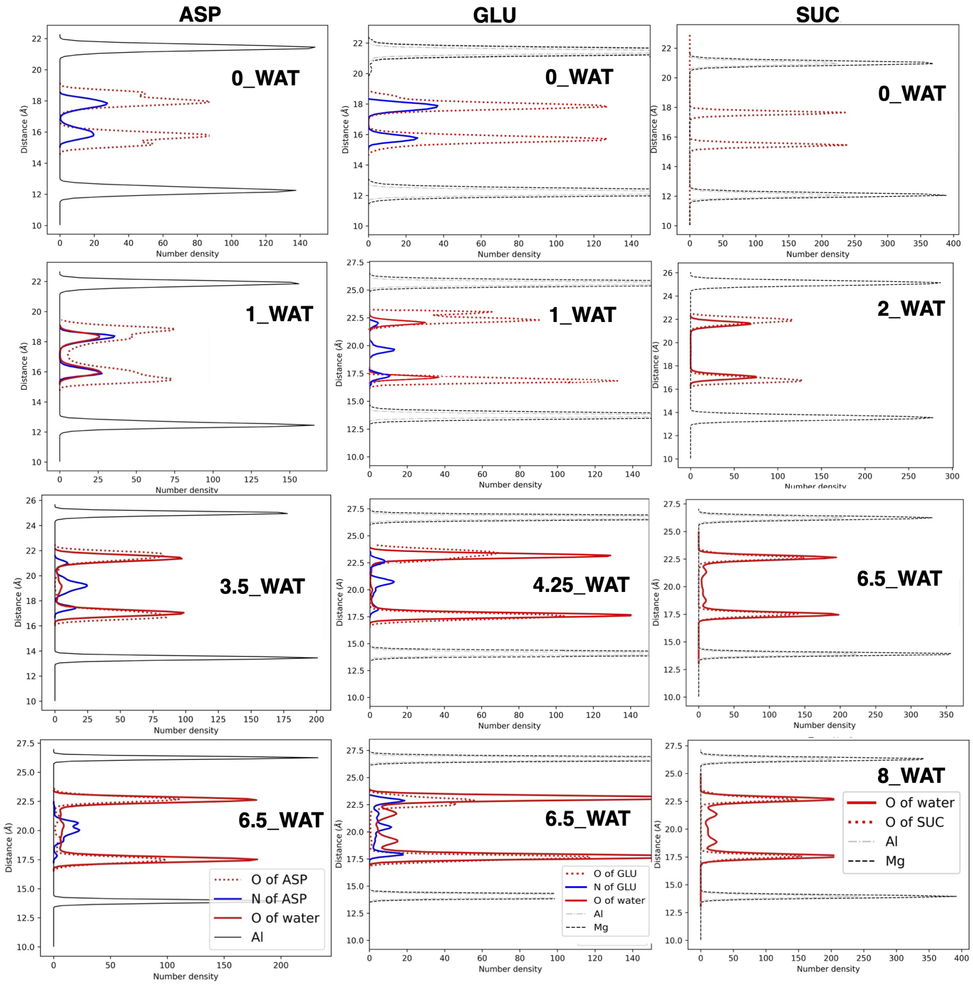

Figure S2: Number densities along the axis perpendicular to the surfaces of relevant atoms in the interlamellar space, shown for the systems at different hydration states for the materials intercalating ASP, GLU and SUC (from left to right).

Conformations

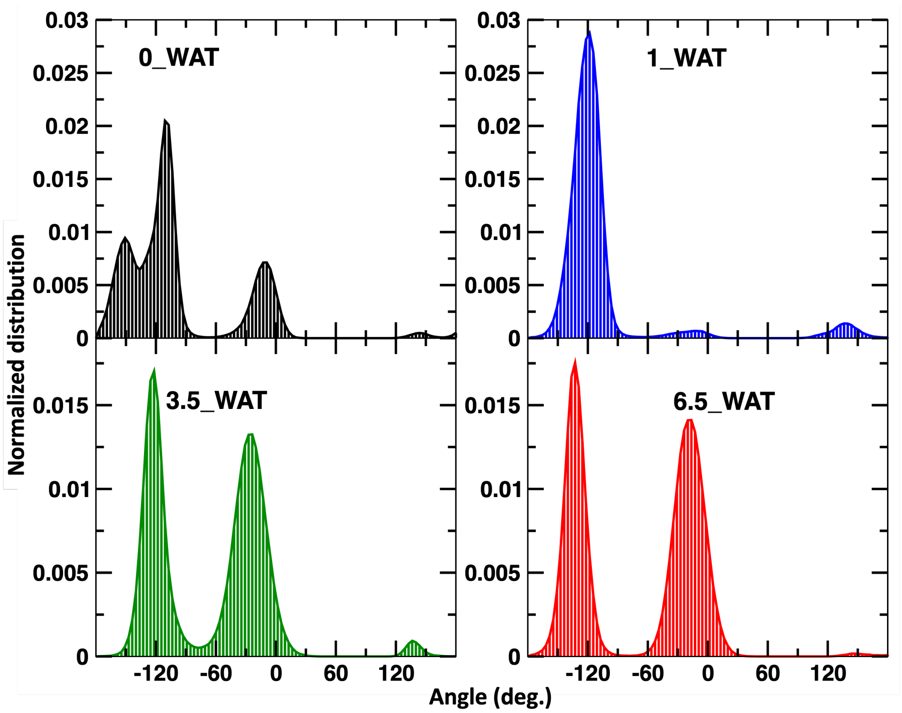

Figure S3: Distribution of the C_α-C_β-C_γ dihedral in the system intercalating aspartate with increasing water content.

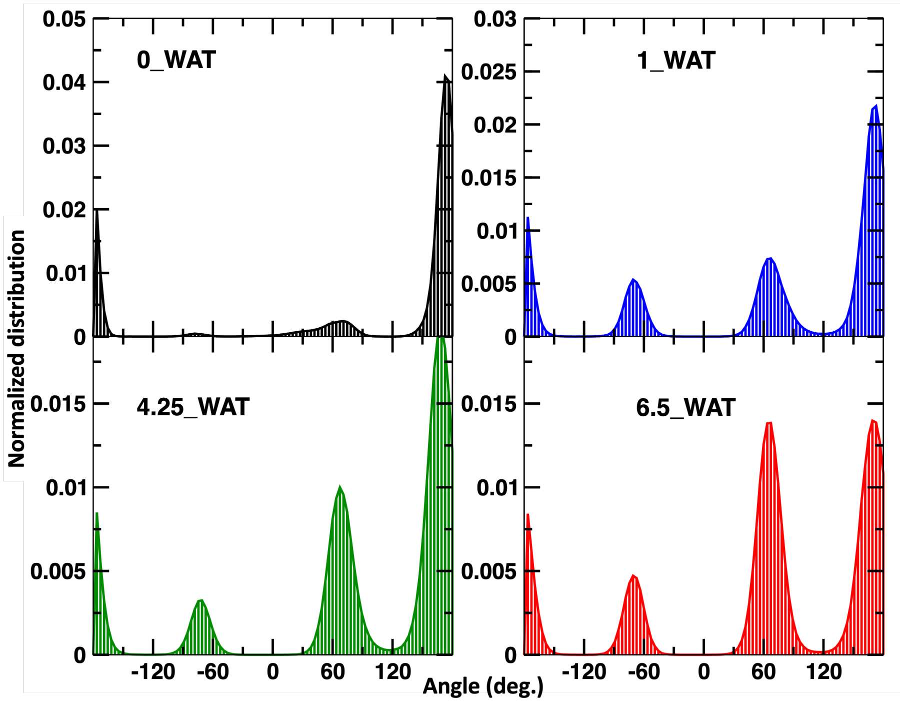

Figure S4: Distribution of the C_α-C_β-C_γ dihedral in the system intercalating glutamate with increasing water content.

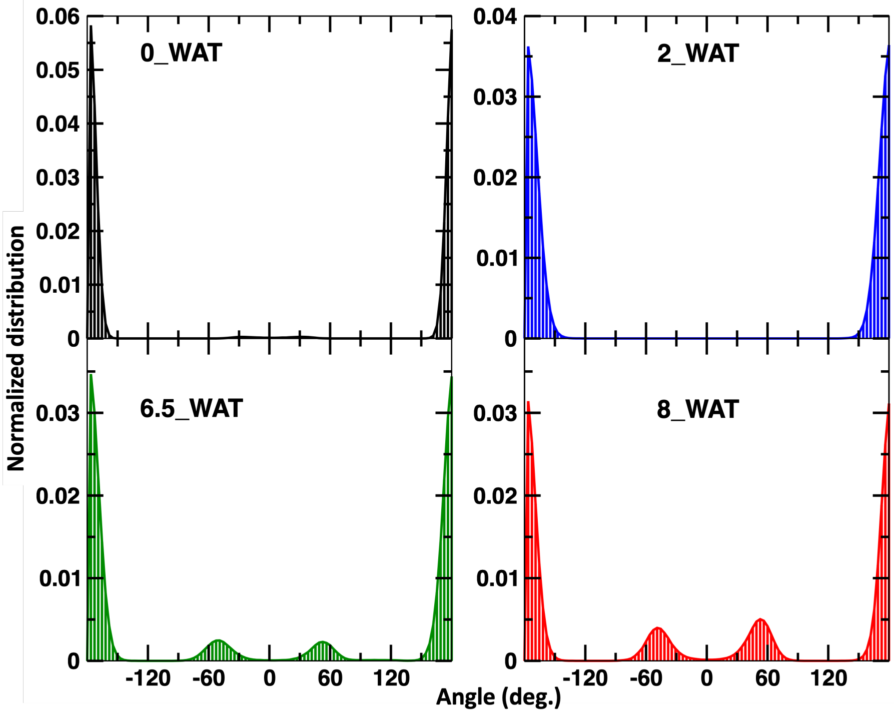

Figure S5: Distribution of the C-C-C-C dihedral in the system intercalating succinate with increasing water content.

References

(1) Cygan, R. T.; Liang, J.-J.; Kalinichev, A. G. Molecular Models of Hydroxide, Oxyhydroxide, and Clay Phases and the Development of a General Force Field. *J. Phys. Chem. B* **2004**, *108*, 1255–1266.

(2) Berendsen, H. J. C.; Postma, J. P. M.; van Gunsteren, W. F.; Hermans, J. In *Intermolecular Forces*; Pullman, B., Ed.; Springer Netherlands: Dordrecht, 1981; Vol. 14; pp 331–342.

(3) Wang, J.; Wolf, R. M.; Caldwell, J. W.; Kollman, P. A.; Case, D. A. Development and Testing of a General Amber Force Field. *J. Comput. Chem* **2004**, *25*, 1157–1174.

(4) Duan, Y.; Wu, C.; Chowdhury, S.; Lee, M. C.; Xiong, G.; Zhang, W.; Yang, R.; Cieplak, P.; Luo, R.; Lee, T.; Caldwell, J.; Wang, J.; Kollman, P. A point-charge force field for molecular mechanics simulations of proteins based on condensed-phase quantum mechanical calculations. *J. Comput. Chem* **2003**, *24*, 1999–2012.

(5) Case, D. et al. AMBER 2016. 2016; University of California, San Francisco.

(6) Humphrey, W.; Dalke, A.; Schulten, K. VMD: Visual Molecular Dynamics. *Journal of Molecular Graphics* **1996**, *14*, 33–38.

(7) Leach, A. R. *Molecular Modelling: Principles and Applications*, 2nd ed.; Prentice Hall: Harlow, England ; New York, 2001.

(8) Kräutler, V.; van Gunsteren, W. F.; Hünenberger, P. H. A Fast SHAKE Algorithm to Solve Distance Constraint Equations for Small Molecules in Molecular Dynamics Simulations. *J. Comput. Chem* **2001**, *22*, 501–508.

(9) Andrea, T. A.; Swope, W. C.; Andersen, H. C. The Role of Long Ranged Forces in Determining the Structure and Properties of Liquid Water. *J. Chem. Phys.* **1983**, *79*, 4576–4584.

(10) Berendsen, H. J. C.; Postma, J. P. M.; van Gunsteren, W. F.; DiNola, A.; Haak, J. R. Molecular Dynamics with Coupling to an External Bath. *J. Chem. Phys.* **1984**, *81*, 3684–3690.
